# Mrs. C. Cumming

**Published:** 1852-07-01

**Authors:** 


### MRS. C. CUMMING.

This long litigated case is, we hope, on the eve of being satisfactorily and amicably arranged. The matter is in the hands of the able Queen's counsel and chancery barrister, Mr. Bethel, and Sir W. Page Wood, who have agreed to adjust the existing differences, and decide what is best for the interest of all parties concerned. No proposition will for one moment be entertained by Mrs. Cumming or her legal advisers which is not based upon the understanding, that the late inquisition of lunacy is to be quashed, and that Mrs. Cumming is to be considered and treated as a sane and rational person. Mrs. Cumming is to enjoy, during the remainder of her life, the whole proceeds of her estate; the expenses of the late commission of lunacy are not to be paid until after her death, Mrs. Cumming agreeing to leave her property to her grandchildren. Should anything unfortunately interfere with the negotiation now pending, the question of Mrs. Cumming's insanity will be submitted to another jury. Should this, alas! be necessary, we have no fear of the result. Mrs. Cumming continues, by order of the Court of Chancery, under the care of Dr. Winslow.

